# Mechanistic Overlaps Between Sleep and Headache Disorders: From Dopaminergic Dysfunction to Neuroinflammation—A Narrative Review

**DOI:** 10.3390/clockssleep8010011

**Published:** 2026-02-27

**Authors:** Miller Martinez, Frank Villarreal, Lourdes M. DelRosso

**Affiliations:** 1 School of Medicine, Universidad Nacional Nueva Granada, Bogota 110111, Colombia; millermatinezj@gmail.com; 2 Department of Neurology, Clinica Ricardo Palma, Lima 15036, Peru; frankvg62@hotmail.com; 3 Department of Family and Community Medicine, University of California San Francisco, Fresno, CA 93710, USA

**Keywords:** headache, migraine, sleep disorders, insomnia, obstructive sleep apnea, restless legs syndrome, circadian rhythms, neuroinflammation, dopamine, iron deficiency

## Abstract

Sleep disorders and primary headache syndromes frequently coexist, and accumulating evidence suggests that this relationship is bidirectional and biologically mediated rather than coincidental. Patients with migraine, tension-type headache, and cluster headache commonly report poor sleep quality, insomnia symptoms, and irregular sleep patterns, while individuals with sleep disorders such as insomnia, obstructive sleep apnea, restless legs syndrome, and narcolepsy experience a higher prevalence, severity, and chronification of headache disorders. This narrative review synthesizes current clinical, epidemiologic, and translational evidence supporting shared neurobiological mechanisms linking sleep and headache disorders. We focus on five major overlapping pathways: dopaminergic dysfunction, iron deficiency, hypothalamic and circadian dysregulation, central sensitization, and neuroinflammation. Evidence from population-based studies, clinical cohorts, neuroimaging, genetic research, and experimental models demonstrates that these mechanisms converge within hypothalamic, brainstem, and trigeminovascular circuits that regulate arousal, pain processing, and homeostasis. Conditions such as insomnia, obstructive sleep apnea, restless legs syndrome, and circadian disruption not only exacerbate headache burden but may act as modifiable risk factors that promote headache onset and progression. Recognizing sleep disorders as integral components of headache pathophysiology has important clinical implications, emphasizing the need for systematic sleep assessment and targeted sleep interventions as part of comprehensive headache management strategies.

## 1. Introduction

A bidirectional relationship has been established between the presence of sleep disturbances and chronic headache disorders [1]. This relationship is evident in the experience of poor sleep quality, insomnia symptoms, and irregular sleep patterns, seen in patients with chronic migraine or chronic tension-type headache, with a prevalence of sleep disruption ranging from 30% to 70%, significantly higher than in the general population [2]. Headache disorders affect approximately 40% of the population, representing the top cause of neurological diagnosis in people older than 5 years. Migraine, tension-type headache, and trigeminal autonomic cephalalgias differ in pain characteristics, anatomical distribution, associated symptoms, and triggers. Migraine affects approximately 15% of the population, whereas tension-type headaches are more prevalent but often underreported, affecting approximately 26% of the population [3]. Overlapping symptoms may contribute to diagnostic uncertainty, particularly in population-based studies [4]. Furthermore, patients diagnosed with sleep disorders such as insomnia, restless legs syndrome, and obstructive sleep apnea also present with higher severity and frequency of various headache syndromes [5,6,7,8]. There is therefore an important differentiation between primary headache disorders, which are characterized by intrinsic neurobiological mechanisms that overlap with sleep regulatory systems, and secondary headaches, which arise from structural, infectious, vascular, or metabolic causes.

The current clinical evidence suggests that sleep disorders and headache syndromes often coexist due to overlap in their underlying neurobiology, particularly within hypothalamic and brainstem circuits that regulate arousal, pain, and homeostasis [9,10], as well as in the pathways involved in circadian regulation [11]. Both in headaches and sleep disorders, there is a significant disruption in neurotransmitter function or regulation, such as serotonin and dopamine [2], as well as disruption in their precursors or cofactors such as iron [12].

Emerging evidence also suggests that inflammatory signaling plays a pivotal role in migraine pathophysiology [13]. Experimental models indicate that the release of neuropeptides initiates a sterile meningeal inflammatory cascade, activating meningeal nociceptors [13]. In parallel, cortical spreading depolarization and neuronal stress can trigger brain parenchymal inflammatory responses and activation of astrocytes and microglia, which then relay pro-inflammatory signals to pain-sensitive meninges, culminating in a headache [13].

This review examines the current state of the art on the bidirectional relationship between primary headaches and sleep disorders, as well as the pathophysiological mechanisms supporting the overlap between both conditions. Secondary headaches were not included because their pathophysiology is largely driven by underlying systemic or focal pathology rather than shared sleep–pain regulatory networks.

## 2. Methods

### 2.1. Article Search

This narrative review was conducted to synthesize clinical, epidemiological, and mechanistic evidence describing the bidirectional relationship between sleep disorders and primary headache syndromes. The review was intentionally designed as a narrative, non-systematic synthesis to integrate findings across sleep medicine, neurology, and pain neuroscience, with emphasis on clinically meaningful patterns and shared pathophysiology rather than quantitative effect estimation.

A comprehensive literature search was performed using PubMed/MEDLINE and EMBASE to identify relevant articles published in English through May 2025. Search terms combined headache-related keywords (e.g., migraine, tension-type headache, cluster headache, trigeminal autonomic cephalalgias) with sleep disorder-specific terms (e.g., insomnia, obstructive sleep apnea, restless legs syndrome, narcolepsy, sleep fragmentation, circadian rhythm), as well as selected mechanistic terms (dopamine, iron deficiency, orexin, hypothalamus, neuroinflammation, central sensitization). Reference lists of key articles were manually reviewed to identify additional relevant studies.

### 2.2. Organization

The literature was organized and synthesized by sleep disorder, including insomnia, obstructive sleep apnea, restless legs syndrome, narcolepsy, circadian rhythm disruption, parasomnias, and sleep-related movement disorders. Within each disorder-specific section, evidence was summarized regarding headache prevalence, headache phenotypes, clinical associations, and proposed biological mechanisms. Mechanistic themes, such as dopaminergic dysfunction, iron dysregulation, hypothalamic and circadian involvement, neuroinflammation, and impaired pain modulation, were discussed within the context of each disorder, rather than as standalone categories, to preserve clinical relevance and reflect real-world comorbidity patterns.

Eligible studies included population-based epidemiologic studies, case–control and cohort studies, clinical series, neuroimaging and genetic investigations, experimental animal studies, and prior narrative or systematic reviews when they contributed to understanding disorder-specific mechanisms or clinical implications. Because of heterogeneity in study design, populations, and outcome measures, formal quality scoring and quantitative synthesis were not performed. Findings were interpreted qualitatively, emphasizing consistency, biological plausibility, and relevance to clinical practice.

This disorder-based organizational approach was chosen to facilitate translation to clinical settings, allowing clinicians to recognize how specific sleep disorders may contribute to headache onset, severity, and chronification, and to identify modifiable sleep-related targets for headache management.

## 3. Results

### 3.1. General Considerations

Clementi et al. studied the relationship between sleep and headache burden in adolescent females (n = 51, mean age 14.9 years). Sleep was assessed using a composite score that incorporated regularity, duration, quality, and efficiency, while headache characteristics were measured through validated questionnaires. The findings showed that poorer multidimensional sleep health was associated with higher headache frequency, intensity, and disability. Specifically, adolescents with worse sleep health reported an average of 12.3 headache days per month, compared with 7.8 days in those with better sleep health. Headache intensity scores were also higher (mean 6.2 vs. 4.7 on a 0–10 scale, *p* < 0.05), and disability measured by the PedMIDAS scale was significantly greater in the poor sleep group (27.4 vs. 14.1, *p* < 0.01) [14].

In another community-based study of 477 participants (61% female, ages 7–84 years) who contributed 4974 diary assessments, sleep quality was a significant predictor of incident headaches. Lower average sleep quality was associated with increased risk of morning (AM) headaches (β = −0.206, 95% CI −0.397 to −0.017, *p* < 0.05). A decrease in sleep quality on the prior day also predicted AM headache onset (β = −0.172, 95% CI −0.305 to −0.039, *p* < 0.05). Changes in subjective energy showed differential effects by time of day, with decreased energy predicting AM headaches (β = −0.145, 95% CI −0.286 to −0.005, *p* < 0.05), while increased energy predicted later-day (PM) headaches (β = 0.157, 95% CI 0.032–0.281, *p* < 0.05). Although stress was associated with headache occurrence, mood and anxiety disorders were not significant predictors after adjustment for clinical and demographic covariates [15].

### 3.2. Insomnia

One retrospective cross-sectional study evaluated sleep and migraine characteristics in 61 new patients at a headache center between August and October 2015. Patients suspected of migraine completed questionnaires that included the Insomnia Severity Index (ISI), headache features, and psychiatric comorbidities. The mean number of migraine headache days per month was 11.6. Only 41% of participants reported sleeping seven or more hours per night, and nearly half (49.2%) screened positive for insomnia (ISI ≥ 15). Insomnia severity did not differ by headache pain intensity, episodic versus chronic migraine, or associated symptoms (nausea, vomiting, photophobia, phonophobia; all *p*  >  0.05). However, musculoskeletal pain (ISI 18.7 vs. 13.8, *p*  =  0.027), depression (rho = 0.610), and anxiety (rho = 0.436) were significantly associated with higher ISI scores. These findings suggest that insomnia is highly prevalent among patients with suspected migraine, independent of the frequency of migraines, and that comorbid pain, depression, and anxiety strongly contribute to sleep disturbance. The authors recommend prioritizing cognitive behavioral therapy for insomnia (CBT-I) referrals, especially for patients with these comorbidities [16].

Consistently, a case–control study conducted in an adult Chinese population (300 migraine patients and 500 age- and sex-matched controls) further confirmed the strong association between insomnia and migraine risk. Participants who reported occasional insomnia had an adjusted odds ratio of 3.51, while those with habitual insomnia reached an odds ratio of 5.42, both highly significant (*p* < 0.001). Overall, the presence of insomnia, regardless of frequency, increased the likelihood of developing migraine by three- to fivefold [1].

Recent population-based research in Egypt found a migraine prevalence of 20.9% among 2533 participants drawn from five regions. Among individuals diagnosed with migraine, 22% reported clinically significant moderate insomnia. Sleep disorders emerged as the most common trigger, reported by 76.9% of migraineurs, surpassing perceived noise (65%) and anxiety (59%). Notably, 46.7% of migraine sufferers experienced severe disability. Multivariate analysis revealed a graded association between insomnia severity and migraine risk: adjusted odds ratios (aORs) increased progressively, from 2.40 for subthreshold insomnia to 4.36 for severe insomnia (95% CI: 1.89–3.05 and 1.97–9.64, respectively). These findings underscore insomnia as not merely a comorbidity but a modifiable risk factor that significantly contributes to the onset, severity, and disability burden of migraine [17].

Additionally, evidence from the prospective Nord-Trøndelag Health Study (HUNT-2 and HUNT-3) highlights that the relationship between migraine and insomnia is bidirectional. In this population-based cohort, individuals with insomnia but no headache were more likely to develop migraine during follow-up. In contrast, migraineurs had nearly a twofold increased risk (OR 1.7) of developing insomnia over 11 years, particularly those with seven or more migraine days per month. The presence of insomnia was also associated with greater migraine pain intensity. Insomnia constitutes a predisposing factor not only for the onset of migraine but also for an increase in pain intensity, clinical impact, and the tendency toward chronification of the disorder [18].

A study conducted among Norwegian nurses with night work evaluated the prevalence of headache and its relationship with different sleep disorders, including insomnia, restless legs syndrome, and shift work disorder. The results showed that insomnia was significantly associated with a higher prevalence of headache, migraine, and chronic headache [19].

### 3.3. Obstructive Sleep Apnea and Headaches

Obstructive sleep apnea (OSA) has a well-established association with “sleep apnea headache,” a specific type of morning headache recognized in the ICHD and often alleviated by OSA treatment. Epidemiologic studies suggest that headaches in general, particularly morning headaches, are more common in patients with OSA than in the general population. However, findings on oxygen desaturation as the sole mechanism are inconsistent. A large U.S. retrospective cohort (TriNetX data, n ≈ 197,000 pairs) found that patients with OSA had a 1.85-fold increased risk of developing migraine compared to matched controls without OSA (HR 1.85, 95% CI 1.79–1.90). This link was consistent across sex, age, race, and BMI subgroups [20]. One study found that 33.6% of patients with OSA reported morning headache versus only 8.9% in non-OSA controls (*p* < 0.01). Symptoms correlated with more severe oxygen desaturation [21]. Another recent study involving 213 patients with OSA found that 24.4% reported headaches. Among those, the breakdown was: tension-type—51.9%; non-specific—42.3%; and migraine-type—5.8%. Key predictors of headache in OSA included dyspnea, fatigue, dizziness, and macroglossia, with dyspnea showing the strongest association (adjusted OR 3.29) [22].

A broader meta-analysis estimated overall headache prevalence in OSA patients at 33%, with specific prevalences of: morning headaches—33%; sleep apnea headaches—25%; tension-type headache: 19%; and migraine—16%. The pooled relative risk of headaches in OSA vs. non-OSA was 1.43 (95% CI 0.92–2.25), indicating a moderate, nonsignificant increase [5].

In addition to migraine and tension-type headache, several studies have also highlighted a strong association between obstructive sleep apnea and cluster headache. Early investigations reported apneic events in more than half of patients with cluster headache, and later studies confirmed that up to 80% exhibit elevated apnea–hypopnea indices. Other studies reported prevalences of 58.3% in cluster headache patients versus 14.3% in control groups, further supporting this link. Hypoxemia during apneic episodes has been proposed as a potential trigger of cluster attacks, consistent with the clinical effectiveness of oxygen therapy in aborting episodes and with the experimental induction of cluster headache under hypoxic conditions. Although some patients improve with continuous positive airway pressure (CPAP), evidence indicates that treating sleep apnea does not invariably prevent cluster attacks. This suggests that both disorders may share a common pathophysiological substrate related to hypothalamic dysfunction, which could explain their coexistence and the circadian timing of cluster headache attacks without implying a direct causal relationship [23].

In a clinical case report, it was documented that, after the initiation of ventilatory support with BiPAP-ST, a patient with chronic treatment-resistant hemiplegic migraine showed a significant reduction in the frequency of crises, going from recurrent and disabling episodes to an average of one event per month. This finding suggests that the correction of central sleep apnea not only optimizes the quality of nocturnal rest but may also constitute an effective therapeutic strategy in the management of refractory headaches [24].

### 3.4. Restless Legs Syndrome

In a large cross-sectional study of 2385 migraine patients and 332 controls without migraine, the prevalence of restless legs syndrome (RLS) was significantly higher in migraineurs (16.9%) compared to controls (9.3%) (adjusted OR 1.83, 95% CI 1.18–2.86; *p* = 0.008). RLS severity was also greater in migraine patients, with a mean severity score of 14.5 compared to 12.0 in controls (*p* = 0.036). Severe to very severe RLS was observed more frequently in migraineurs (14.1%) than in non-migraine controls (3.2%). Sleep quality, measured by the Pittsburgh Sleep Quality Index (PSQI), was markedly poorer among migraine patients, especially those with comorbid RLS. Poor sleep quality (PSQI ≥ 6) was present in 64.3% of migraineurs with RLS compared to 51.3% without RLS (*p* < 0.001). Global PSQI scores were significantly higher in migraineurs with RLS (7.4 ± 3.7) than those without RLS (6.3 ± 3.6; *p* < 0.001), indicating more fragmented and insufficient sleep. RLS in migraineurs was independently associated with both poor sleep quality and higher insomnia severity [7].

In a case–control study of 47 patients with restless legs syndrome (RLS) and 47 age- and sex-matched controls, patients with RLS exhibited significant clinical differences compared with controls. They had worse sleep quality (PSQI 11.4 ± 4.4 vs. 7.1 ± 4.2, *p* < 0.001), had higher anxiety (42.7 ± 9.2 vs. 36.8 ± 7.3, *p* = 0.004) and depression scores (45.3 ± 13.0 vs. 38.7 ± 9.8, *p* = 0.021), and were more frequently treated with dopaminergic agonists (31.9% vs. 2.1%, *p* < 0.001) and antidepressants (40.4% vs. 19.1%, *p* = 0.031). No significant differences were found in BMI, sex distribution, or most other medication use [6].

Notably, the lifetime prevalence of migraine was significantly higher in the RLS group compared with controls (53.2% vs. 25.5%, *p* = 0.005). Active migraine without aura showed the strongest association (40.4% vs. 12.8%, *p* = 0.001), with an adjusted odds ratio of 2.7. No significant relationship was observed between RLS and inactive migraine or migraine with aura. Within the RLS cohort, those with migraine reported poorer sleep quality than those without migraine (PSQI > 5: 100% vs. 80.9%, *p* = 0.038), though there were no differences in RLS severity, anxiety, depression, BMI, or dopaminergic treatment [6].

Yang et al. investigated the association between restless legs syndrome (RLS) and primary headaches in a cohort of 31 RLS patients and 31 matched controls. They reported that 61% of RLS patients experienced recurrent headaches compared with 32% of controls (*p* < 0.05). The prevalence of migraine was also higher in the RLS group (39% vs. 16%), while tension-type headache showed a nonsignificant trend (22% vs. 16%). Importantly, RLS patients with comorbid headache had poorer sleep quality and greater daytime fatigue scores than RLS patients without headache, indicating an additive negative impact on quality of life [25].

In the Women’s Health Study, which followed over 31,000 women, migraine was found to be associated with an increased risk of restless legs syndrome (RLS). Among the 6857 women reporting migraine at baseline or during follow-up, 14.5% had RLS compared with 11.2% of women without migraine. After adjusting for multiple covariates, migraine was linked to a 22% higher odds of RLS (OR = 1.22; 95% CI, 1.13–1.32). This association was consistent for migraine with aura (OR = 1.27; 95% CI, 1.10–1.48), migraine without aura (OR = 1.24; 95% CI, 1.09–1.40), and new reports of migraine during follow-up (OR = 1.30; 95% CI, 1.10–1.54). By contrast, women with only a prior history of migraine did not show a significant association (OR = 1.11; 95% CI, 0.96–1.28). These findings suggest that active migraine, regardless of aura status, is associated with a higher prevalence of RLS, while inactive or past migraine does not confer an increased risk [26].

Iron deficiency represents a key mechanistic overlap between restless legs syndrome (RLS) and migraine, given its role in dopaminergic neurotransmission and neurovascular regulation. Several studies demonstrate that migraine patients, particularly women, have a higher prevalence of iron deficiency anemia (IDA) compared to controls, with menstrual migraine showing the strongest association [10]. Low ferritin levels are inversely correlated with migraine severity and disability scores, suggesting that reduced iron stores exacerbate headache burden [27]. Importantly, iron supplementation has been shown to significantly reduce migraine frequency, duration, and intensity in patients with confirmed IDA, highlighting its therapeutic potential [28]. Since iron deficiency is also central to the pathophysiology of RLS, these findings reinforce the hypothesis that iron dysregulation is a shared pathway that may partly explain the frequent comorbidity of RLS and migraine.

RLS pathophysiology has long been associated with dopamine dysfunction [29]. This can be a key mechanism underlying the coexistence of RLS and migraine. In migraine with aura, variants in dopamine-related genes, including dopamine beta-hydroxylase (DBH), dopamine D2 receptor (DRD2), and the dopamine transporter (SLC6A3), have been associated with altered susceptibility, supporting the hypothesis that dopaminergic signaling contributes to migraine pathogenesis [30]. These findings complement well-established evidence of dopaminergic abnormalities in RLS, where impaired hypothalamic A11 dopamine neuron activity and disrupted striatal dopamine transmission underlie motor restlessness and sleep disruption [31]. Together, the presence of dopaminergic dysfunction in both conditions suggests a common biological substrate that may explain their frequent coexistence. Clinically, this overlap is further supported by the partial responsiveness of both disorders to dopamine-modulating therapies [32,33].

### 3.5. Narcolepsy

Narcolepsy is a disorder characterized by excessive daytime sleepiness that is not due to alterations of the circadian rhythm. Several studies have reported a high prevalence of migraine in narcoleptic patients, reaching figures of up to 37%. Likewise, cases have been documented in which tension-type headache constitutes the main complaint in this group of patients.

The possible causal relationship has been linked to dysregulation of brainstem structures, including the periaqueductal gray matter, the dorsal raphe nucleus, and the locus coeruleus. These regions play a fundamental role in the transmission and modulation of pain in migraine, and are also involved in promoting wakefulness and regulating the sleep–wake transition.

A central mechanism involves the orexinergic system. Orexins A and B, neuropeptides synthesized in the lateral hypothalamus, participate in the maintenance of the wake state. Under normal conditions, orexin levels are higher during wakefulness. In narcolepsy, a dysfunction of the hypothalamic orexinergic system has been evidenced with loss of orexin-producing neurons, which not only contributes to the pathophysiology of the sleep disorder but has also been implicated in the pathophysiology of migraine [18].

### 3.6. Bruxism, Sleep, and Headaches

In a cross-sectional study of 180 patients with temporomandibular disorders (TMDs), the prevalence of five comorbid conditions, migraine, chronic fatigue syndrome (CFS), irritable bowel syndrome (IBS), interstitial cystitis (IC), and restless legs syndrome (RLS), was evaluated. The findings demonstrated that migraine (54.6% vs. 28.8%) and CFS (19.0% vs. 5.1%) were significantly more prevalent in patients with myofascial TMD compared to those with nonmyofascial TMD, supporting the hypothesis that these conditions may share overlapping mechanisms of central sensitization. Similarly, IBS and IC were somewhat more frequent in the myofascial subgroup, though the differences did not reach the same level of statistical strength.

In contrast, RLS showed no meaningful difference between groups, with a prevalence of 16.5% in myofascial TMD and 16.9% in nonmyofascial TMD (*p* = 0.94). Logistic regression confirmed the absence of an association between RLS and TMD subtype (OR 0.66, 95% CI 0.25–1.75). These findings suggest that, unlike migraine and CFS, RLS does not appear to share the same central sensitivity syndrome characteristics in this patient population [34].

### 3.7. Headaches and Sleepwalking

An observational study with 100 patients diagnosed with sleepwalking analyzed the relationship between this parasomnia, the perception of pain during nocturnal episodes, and the prevalence of headaches. The results showed that people with sleepwalking present a higher risk of suffering from headaches and migraines compared to the general population. It is noteworthy that, although migraine symptoms are relevant during the day, during severe nocturnal episodes, patients do not perceive pain, even in situations of significant injuries such as fractures, which suggests a dissociation in the mechanisms of pain perception during sleep [35].

Mechanistically, serotonergic neurons originating in the median and dorsal raphe nuclei play a central role in both nociceptive modulation and sleep–wake regulation, and alterations in serotonin synthesis, metabolism, and receptor signaling have been consistently documented in migraine. Dysregulation of these pathways may impair arousal stability during slow-wave sleep, thereby facilitating parasomnic behaviors, while simultaneously enhancing trigeminovascular excitability and headache susceptibility [1]. Furthermore, the reciprocal interactions between serotonergic and orexinergic systems suggest that disruption of this regulatory network may contribute to the co-occurrence of migraine and sleepwalking [36].

### 3.8. Electronic Use, Sleep, and Headaches

One multicenter, cross-sectional study (n = 400; mean age 27.6 years; 65.8% female) examined the association between mobile phone overuse and migraine outcomes. Participants were divided into high-mobile-phone-use (HMPUG) and low-mobile-phone-use (LMPUG) groups using the Mobile Phone Problematic Use Scale. Both groups were assessed for migraine disability (MIDAS), pain severity (VAS), sleep quality (PSQI), daytime sleepiness (ESS), and quality of life (24 h MQoLQ) [33]. VAS was higher in HMPUG (VAS 5.9 ± 2.5) vs. LMPUG (VAS 5.1 ± 2.7; *p* = 0.009). LMPUG reported more frequent use and better relief, whereas HMPUG showed reduced medication efficacy (*p* = 0.007). Severe disability was more frequent in LMPUG, showing a negative correlation between mobile phone use and disability (r = –0.124; *p* = 0.030) [33]. Poor sleep was significantly more common in HMPUG (PSQI; *p* < 0.001), with a positive correlation between phone overuse and poor sleep quality (r = 0.301). HMPUG had higher normal daytime sleepiness, but correlations with MPPUS were weak and inconsistent. HMPUG reported significantly higher scores on the 24 h MQoLQ (*p* < 0.001), suggesting a paradoxical improvement in perceived quality of life despite worse pain and sleep. In summary, excessive smartphone use in migraine patients was associated with greater pain intensity, poor sleep quality, and reduced medication effectiveness, but not with increased migraine frequency or disability. Interestingly, higher phone use correlated with better reported quality of life, possibly due to social connectedness and coping via smartphone use [37].

### 3.9. Medication Effect

Medications to treat primary headaches can have implications for sleep. Among the treatment options for acute migraine attacks, the lipophilic triptans such as eletriptan, zolmitriptan, and rizatriptan have been associated with greater post-treatment somnolence compared with less lipophilic agents such as sumatriptan, almotriptan, and naratriptan [38]. In contrast, nonsteroidal anti-inflammatory drugs (NSAIDs) appear to have minimal impact on sleep architecture [38]. To date, there is no data on potential sleep-related side effects of Rimegepant, an oral calcitonin gene-related peptide (CGRP) receptor antagonist used for both acute and preventive migraine treatment [39].

Several medications used for long-term migraine prevention may interfere with sleep. Beta-blockers, particularly propranolol, are associated with an increased risk of insomnia, especially when administered at night [40]. Tricyclic antidepressants such as amitriptyline are associated with increased total sleep time, reduced rapid eye movement (REM) sleep, and increased periodic limb movements during sleep [41]. Among antiepileptic agents, topiramate, lamotrigine, and valproic acid may affect sleep architecture [42].

In summary, the relationship between sleep disorders and headache is heterogeneous and varies across headache phenotypes, reflecting differences in underlying neurobiology and clinical expression. Specific headache types, including migraine, tension-type headache, and trigeminal autonomic cephalalgias, demonstrate preferential associations with particular sleep disorders and mechanistic pathways. Table 1 provides an overview of major primary headache types and the proposed shared pathophysiological mechanisms that may explain their simultaneous presentation with sleep disorders, drawing on evidence from clinical, experimental, and translational studies.

## 4. Discussion

**Figure 1:**
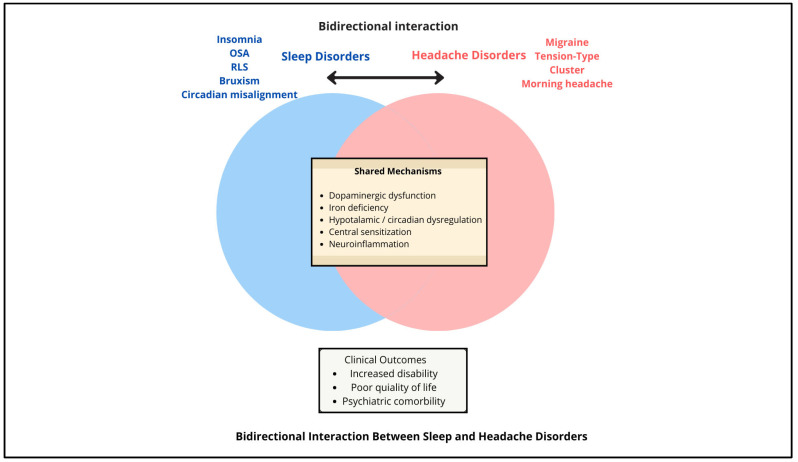
Bidirectional interaction between sleep and headache disorders, with the shared mechanistic overlap.

The relationship between sleep and headache disorders is best understood through the lens of shared neurobiological mechanisms that establish a bidirectional cycle. As depicted in Figure 1, five overlapping pathways, dopaminergic dysfunction, iron deficiency, hypothalamic/circadian dysregulation, central sensitization, and neuroinflammation, play a central role in linking conditions such as migraine, restless legs syndrome (RLS), insomnia, and obstructive sleep apnea (OSA).

The hypothalamus serves as a hub linking sleep, circadian rhythms, and headache pathophysiology [9]. Cluster headache, for example, exhibits strict circadian timing, with functional imaging implicating posterior hypothalamic activation [43]. Similarly, RLS symptoms worsen at night in alignment with circadian fluctuations of dopamine and iron [44]. Insomnia and circadian misalignment further exacerbate migraine chronification, underscoring the role of hypothalamic dysfunction and disrupted circadian neurochemistry as common drivers of both sleep and headache disorders [11].

The relationship between sleep and pain modulation could be explained by the fact that the orexinergic system projects to the periaqueductal gray, involved in descending pain modulation [45]. In mice, microinjection of orexin A into the posterior hypothalamus inhibited trigeminal nociceptive response evoked by dural electric stimulation, while orexin B increased it [46]. This relationship was studied by Finan and colleagues, who demonstrated hyperalgesic effects of sleep deprivation in patients with chronic pain [47].

Neuroinflammation also represents a unifying pathway. Elevated levels of pro-inflammatory cytokines, including IL-6 and TNF-α, have been documented in both migraine and primary sleep disorders such as insomnia and OSA [1,13,48]. Glial activation within trigeminovascular and hypothalamic circuits further contributes to pain amplification and sleep disturbance, closing the loop between sleep dysregulation and headache burden [49,50].

The glymphatic system, responsible for neuromodulation and waste clearance in the CNS, is implicated in pain generation in migraine and trigeminal autonomic headaches [51]. Neuroinflammation is a proposed mechanism, with astrocytes releasing pro-inflammatory molecules such as TNF-α (tumoral necrosis factor α), IL-1β (Interleukine-1β) and HIF-1α (hypoxia-induced factor-1α) in response to reactive oxygen species [52]. A case–control study of 100 migraine patients evaluated glymphatic function via DTI-ALPS MRI protocol and correlated it with the Pittsburgh Sleep Quality Index. Lower DTI-ALPS scores (reduced glymphatic clearance) were associated with higher migraine frequency in poor sleepers [53].

In terms of RLS, emerging evidence underscores overlapping neurobiological pathways that may underlie the frequent comorbidity with migraine. Both conditions appear to be mediated at least in part by dopaminergic dysfunction, where patients with migraine exhibit hypersensitivity of D2-like receptors in brainstem regions [31], while RLS is characterized by impaired dopaminergic signaling in the basal ganglia and hypothalamic A11 neurons [54]. Additionally, brain iron deficiency—commonly implicated in RLS—is also found in some migraine patients and may exacerbate dopaminergic dysregulation [55]. Sleep disruption, prevalent in both disorders, likely contributes via alterations in circadian neurochemical rhythms and impaired pain modulation pathways.

The CKIδ gene has been linked to migraine and circadian rhythm disorders [56]. A study identified two families with CKI gene mutations showing delayed sleep phase syndrome and migraine [57]. The same study found that CKI-altered mice showed increased pain susceptibility and cortical spreading depression when exposed to nitroglycerin to mimic migraine symptoms.

Genetic studies hint at shared susceptibility loci, such as the region on chromosome 14q21 linked to familial forms of both RLS and migraine. Finally, central sensitization, well recognized in chronic migraine via trigeminocervical convergence, and structural CNS alterations observed in imaging studies further reinforce the notion of converging pathophysiological processes.

A case–control study on patients with bruxism and sleep apnea with or without migraine found higher frequency of mixed bruxism in migraine patients. Respiratory episode duration correlated with incidence of bruxism episodes, suggesting a shared mechanism [58].

Recent epidemiological data indicate that sleep disorders are not merely comorbid conditions but significant contributors to headache pathophysiology. Case–control and population-based studies have demonstrated a strong, graded association between insomnia and migraine, while the HUNT study confirmed a bidirectional relationship over long-term follow-up. Similarly, the high prevalence of obstructive sleep apnea among patients with cluster headache suggests shared mechanisms involving hypoxemia and hypothalamic dysfunction. Taken together, these findings position insomnia and sleep apnea as modifiable risk factors that can increase headache burden and facilitate chronification, emphasizing the importance of systematically incorporating sleep assessment into headache management.

Sleep disorders and primary headache syndromes frequently intersect through overlapping neurobiological pathways rather than through isolated or coincidental mechanisms. Multiple lines of evidence suggest that disturbances in neurotransmitter regulation, circadian and hypothalamic function, inflammatory signaling, and pain modulation contribute to both sleep disruption, headache burden, and disability [59,60]. To integrate these converging pathways and their clinical relevance, Table 2 summarizes the principal shared mechanisms linking sleep disorders and headache syndromes, the supporting evidence in both domains, and the associated clinical implications and modifiable risk factors. A potential limitation of this review is the inconsistent differentiation between migraine and other headache phenotypes, which may have introduced diagnostic heterogeneity.

## 5. Conclusions

Sleep and headache disorders are closely intertwined through shared neurobiological pathways that extend beyond simple comorbidity. Evidence across epidemiologic, clinical, genetic, and experimental studies indicates that disturbances of sleep continuity, circadian regulation, and arousal systems can amplify headache susceptibility, severity, and chronification, while recurrent headache further disrupts sleep and pain-modulating networks. Disorders such as insomnia, obstructive sleep apnea, restless legs syndrome, and narcolepsy interact with headache syndromes through converging mechanisms involving hypothalamic and brainstem dysfunction, dopaminergic and iron-related pathways, neuroinflammation, and central sensitization. Recognizing sleep disorders as integral contributors to headache pathophysiology reframes them as modifiable targets rather than secondary comorbidities. Integrating systematic sleep assessment and targeted sleep interventions into headache management has the potential to reduce disease burden, improve treatment response, and interrupt the bidirectional cycle linking sleep disruption and chronic headache.

## Figures and Tables

**Table 1:** Primary headache types and proposed pathophysiological mechanisms underlying co-occurrence with sleep disorders.

Type of Primary Headache	Primary Mechanistic Domain	Shared Pathophysiological Mechanisms with Sleep Disorders
**Tension-Type Headache**	Inflammatory	Sleep deprivation and intermittent hypoxia show elevated IL-1β, IL-6, TNF-α.
	Central Sensitization	Central sensitization, sleep deprivation linked to hyperalgesic effects in chronic pain patients.
**Migraine**	Genetic	Mutations of Gen CKIδ are present in two families with circadian disorders and migraine.
	Inflammatory	Elevated IL-1β, IL-6, TNF-α.
	Central Sensitization	Orexin A reduces trigeminal nociceptive response. Sleep deprivation is linked to hyperalgesia in patients with chronic pain.
	Peripheral Sensitization	Sleep apnea is associated with increased cervical pain in migraine patients.
	Clinical	Reduced glymphatic clearance in migraine patients with poor sleep quality.
**Trigeminal Autonomic Cephalalgias**	Central Sensitization	Orexin A reduces trigeminal nociceptive response. Sleep deprivation is linked to hyperalgesia in patients with chronic pain.

**Table 2:** Shared mechanisms, clinical pathways, and modifiable risk factors linking sleep disorders and headache syndromes.

Mechanism/Disorder	Pathophysiological Basis	Evidence in Sleep Disorders	Evidence in Headache	Clinical Implications/Modifiable Factors
Dopaminergic dysfunction	Altered dopaminergic signaling in hypothalamus, basal ganglia, and brainstem	RLS: impaired A11 dopaminergic neurons and altered striatal transmission	Migraine: hypersensitivity of D2-like receptors; genetic variants DBH, DRD2, and SLC6A3	Dopamine agonists improve RLS; dopamine-targeted migraine therapies are under study
Iron deficiency	Iron is essential for dopamine synthesis and neurovascular stability	RLS strongly linked to low ferritin/brain iron deficiency	Menstrual migraine and increased severity with iron deficiency	Ferritin screening; iron supplementation reduces RLS symptoms and migraine burden
Circadian/hypothalamic dysregulation	Hypothalamic nuclei regulate sleep, circadian rhythms, and pain	RLS worsens at night; insomnia and circadian misalignment are common	Cluster headache shows circadian periodicity; migraine chronification worsens with irregular sleep	Behavioral interventions (CBT-I, light therapy), circadian realignment, and sleep hygiene
Neuroinflammation	Activation of astrocytes and microglia; release of IL-6, TNF-α, and CGRP	OSA and insomnia are linked to systemic and CNS inflammation	Migraine with PET/MRI evidence of meningeal and parenchymal inflammation	Targeted therapies: anti-CGRP agents; lifestyle/anti-inflammatory strategies (exercise, diet, stress control)
Central sensitization	Enhanced excitability of nociceptive pathways; impaired pain modulation	Insomnia, TMD, and fragmented sleep promote sensitization	Chronic migraine, TMD, and facial pain syndromes involve sensitization	Early treatment of insomnia/OSA may prevent chronification; multidisciplinary pain management
Sleep fragmentation/insomnia	Hyperarousal and fragmented sleep reduce pain thresholds	Insomnia prevalence 30–50% in the general population	Increases migraine onset and chronification	CBT-I, sleep hygiene, and targeted pharmacotherapy
Neuromuscular factors (bruxism, TMD)	Parafunctional jaw activity increases myofascial strain	Poor sleep quality and musculoskeletal pain in TMD	Migraine and CFS are more common in TMD patients	Stress reduction, splints, and mandibular physiotherapy
Excessive electronic device overuse	Circadian disruption via blue light and overstimulation	Overuse is associated with poorer sleep quality	Increased migraine pain severity and intensity	Limiting nighttime use, blue light filters, and digital hygiene
Narcolepsy	Loss of hypothalamic orexin-producing neurons → orexinergic system dysfunction	Excessive daytime sleepiness, cataplexy, and orexin deficiency were documented	Migraine prevalence up to 37% in narcoleptics; tension-type headache may predominate	Possible therapeutic role of orexin modulation: routine headache assessment in narcoleptics
Sleepwalking	Parasomnia with dissociation of sleep–wake circuits and pain perception	Patients with sleepwalking do not perceive pain during episodes, even with severe injuries	Higher risk of headaches and migraine compared to the general population	Clinical relevance in differential diagnosis and injury prevention during nocturnal episodes

## Data Availability

No new data were created or analyzed in this study. Data sharing is not applicable to this article.

## Abbreviations

The following abbreviations are used in this manuscript:

AHI	Apnea–Hypopnea Index
AM	Morning
aOR	Adjusted Odds Ratio
BMI	Body Mass Index
BiPAP	Bilevel Positive Airway Pressure
CAP	Cyclic Alternating Pattern
CBT-I	Cognitive Behavioral Therapy for Insomnia
CFS	Chronic Fatigue Syndrome
CI	Confidence Interval
CKIδ	Casein Kinase I Delta
CPAP	Continuous Positive Airway Pressure
CSD	Cortical Spreading Depolarization
CNS	Central Nervous System
CRP	C-Reactive Protein
DBH	Dopamine Beta-Hydroxylase
DRD2	Dopamine D2 Receptor
DTI-ALPS	Diffusion Tensor Imaging–Analysis Along the Perivascular Space
EEG	Electroencephalography
ESS	Epworth Sleepiness Scale
GABA	Gamma-Aminobutyric Acid
HIF-1α	Hypoxia-Inducible Factor 1 Alpha
HR	Hazard Ratio
IC	Interstitial Cystitis
ICHD	International Classification of Headache Disorders
IDA	Iron Deficiency Anemia
IL	Interleukin
ISI	Insomnia Severity Index
MIDAS	Migraine Disability Assessment Scale
MRI	Magnetic Resonance Imaging
NMDA	N-Methyl-D-Aspartate
OR	Odds Ratio
OSA	Obstructive Sleep Apnea
PAC1	Pituitary Adenylate Cyclase-Activating Polypeptide Type 1 Receptor
PedMIDAS	Pediatric Migraine Disability Assessment Scale
PET	Positron Emission Tomography
PM	Afternoon/Evening
PSG	Polysomnography
PSQI	Pittsburgh Sleep Quality Index
RDI	Respiratory Disturbance Index
REM	Rapid Eye Movement
RLS	Restless Legs Syndrome
ROS	Reactive Oxygen Species
SLC6A3	Dopamine Transporter Gene
TMD	Temporomandibular Disorder
TNF-α	Tumor Necrosis Factor Alpha
VAS	Visual Analog Scale
